# The Unfamiliar Case of COVID-19 Induced Cerebral Venous Sinus Thrombosis in a Pediatric Patient

**DOI:** 10.7759/cureus.17209

**Published:** 2021-08-16

**Authors:** Priyanka Anvekar, Petras Lohana, Abdul mukhtadir Kalaiger, Syed R Ali, Rohit S Galinde

**Affiliations:** 1 Medicine and Surgery, MGM Medical College and Hospital, Mumbai, IND; 2 Internal Medicine, Liaquat University of Medical and Health Sciences Hospital, Karachi, PAK; 3 Cardiology, Mayo Clinic, Rochester, USA; 4 Internal Medicine, Civil Hospital, Dow University of Health Sciences, Karachi, PAK; 5 Radiodiagnosis, Lata Mangeshkar Hospital, Nagpur, IND

**Keywords:** covid-19, cerebral venous sinus thrombosis (cvst), pediatric stroke, therapeutic anticoagulation, covid-19 associated coagulopathy

## Abstract

The SARS-CoV-2 virus responsible for COVID-19 infection has affected the world from the end of 2019 with pulmonary and extrapulmonary manifestations. Hematologic complications are a rare but severe complication of the COVID-19 infection. There have been very few cases reported in the past showing thrombotic complications in the pediatric age group. We present a case of a 12-year-old male child showing cerebral venous sinus thrombosis (CVST) who tested positive for COVID-19 at the same time. We highlight the potential of this complication in the pediatric age group and discuss the treatment, which is an infrequent phenomenon.

## Introduction

In late 2019, the entire world was affected by SARS-CoV-2, which was soon declared as a pandemic in early 2020. Even though many extrapulmonary manifestations of COVID-19 infection and coagulopathy have been commonly seen [1,2], there has been not much mention in the literature about COVID-19 related cerebral venous sinus thrombosis (CVST) in the pediatric age group. We present a case of a 12-year-old boy showing CVST with concomitant SARS-CoV-2 infection successfully treated with anticoagulants.

## Case presentation

**Figure 1:**
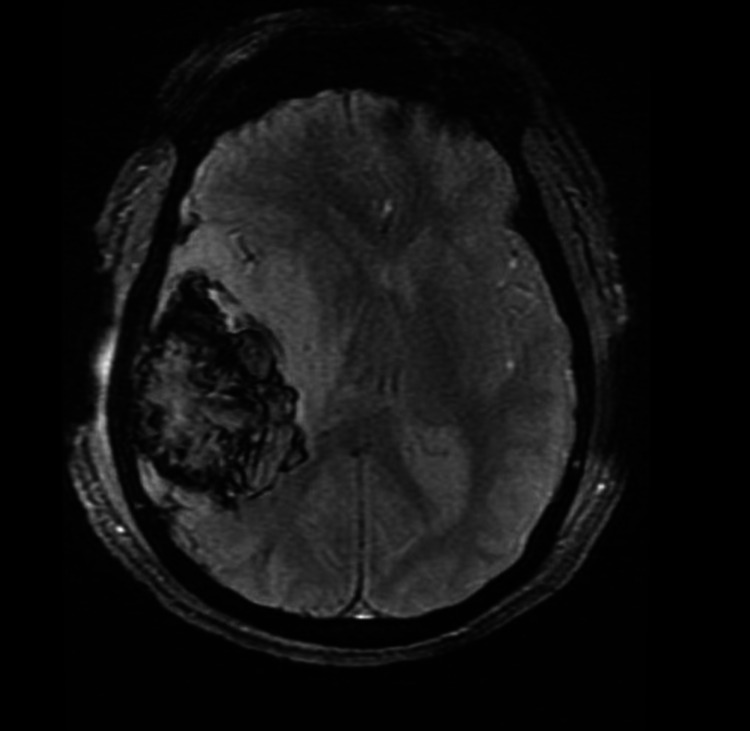
MRI of the brain suggestive of a right temporoparietal large hemorrhagic lesion

A 12-year-old right-handed male child presented to the emergency department due to altered mental status (AMS). He further complained of low-grade fever, body pains, cough, and headache for five days. He described his headache as worse in the morning and associated with nausea and vomiting for the past three days. He denied any significant past medical or surgical history. On examination, he was afebrile, oriented but complained of severe headache. His pulse was 89/min, his respiratory rate was 15/min, his blood pressure was 136/88mmHg, and he maintained a saturation of 98%. A neurology consult was given. Cranial nerve examination was normal. Fundoscopic investigation revealed papilledema. Initial MRI of the brain was suggestive of a right temporoparietal large hemorrhagic lesion (Figure 1).

**Figure 2:**
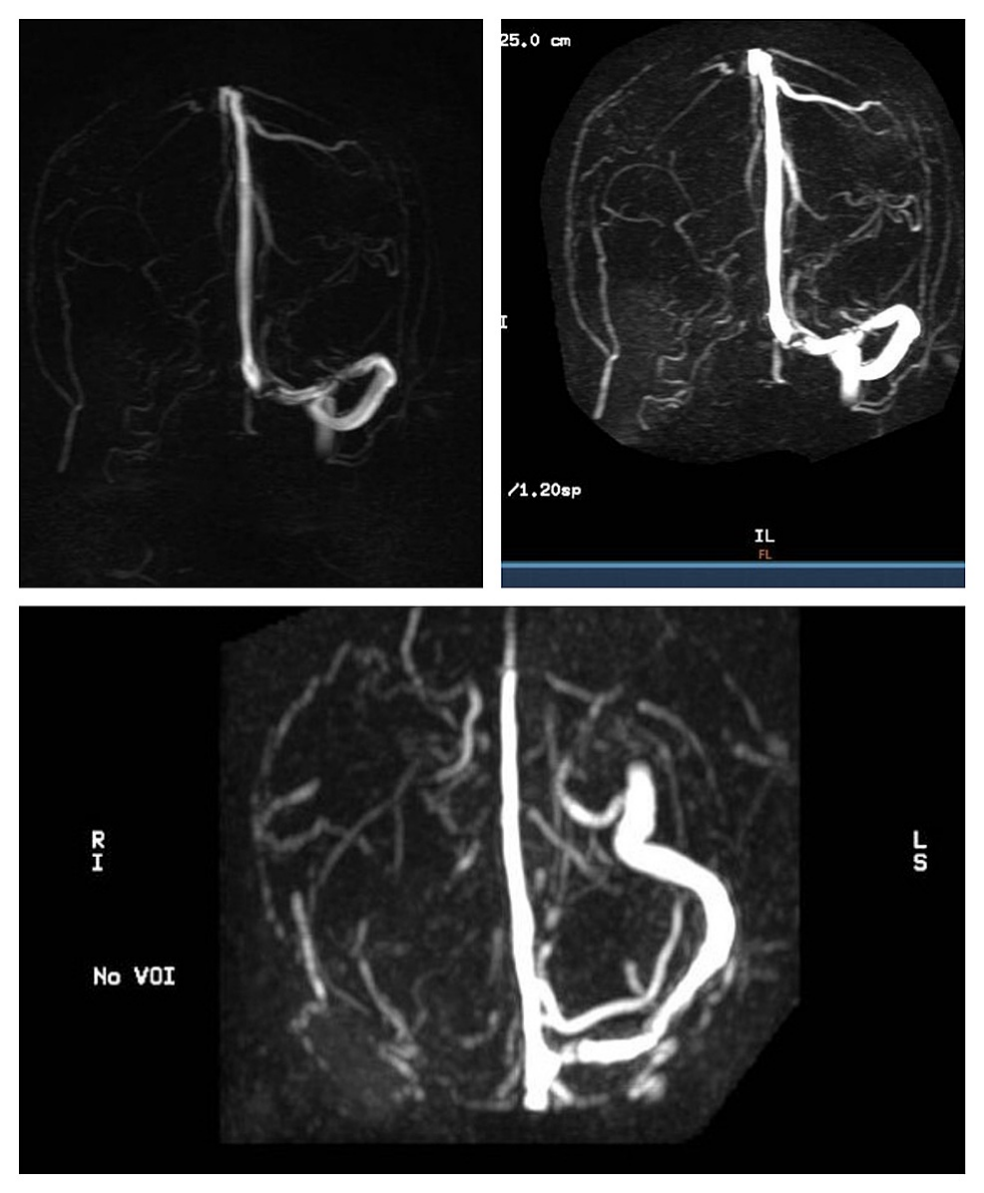
MRI of the brain with MRV revealed massive cerebral venous thrombosis involving sigmoid, lateral, and jugular venous sinuses MRV: Magnetic resonance venography

MRI of the brain with magnetic resonance venography (MRV) revealed massive cerebral venous thrombosis involving sigmoid, lateral, and jugular venous sinuses (Figure 2).

Nasal swab polymerase chain reaction (PCR) was positive for COVID-19. He denied any family history of thrombotic disease or usage of any medications leading to the prethrombotic condition. Table 1 shows the investigation done at the time of admission.

**Table 1:** Investigations done at the time of admission INR: International normalized ratio

Investigation	Laboratory values	Range
White blood count (k/mm3)	8.6	4.8-10.8
Hemoglobin (g/dL)	11.2	11-13
Prothrombin time (sec)	10.9	9.8-12
Activated prothrombin thromboplastin time (sec)	33	21-41
International normalized ratio (INR)	1.04	1.0
Fibrinogen (gm/dL)	289	108-400
D-dimer (mg/L)	1.28	<0.5
Platelets (x 10^9^/L)	366	150-450

Further, hypercoagulability profiles including protein C, protein S, homocysteine, antithrombin III, factor V Leiden, anticardiolipin antibodies, prothrombin gene mutation within normal range. Factor VIII activity was found abnormal at 248% (normal range 50-150%). He was admitted to the ICU and was started on enoxaparin 1mg/kg twice a day for anticoagulation. His neurological status improved through his hospital stay. The patient was discharged on anticoagulation treatment for 90 days. Monthly follow-up was normal and managed conservatively.

## Discussion

Very limited studies have been published showing an association between COVID-19 and CVST in the pediatric age group [3]. The SARS-CoV-2 infection has been linked to the development of pro-thrombotic conditions in patients. One of the proposed mechanisms is that the high level of degradation products of D-dimer, fibrinogen, and fibrin due to the infection causes the hypercoagulable state [3]. The SARS-CoV-2 infection can also decrease the availability of angiotensin-converting enzyme (ACE2) by interacting with its receptors, further contributing to thrombus formation [4]. The activation of the inflammatory cascade by the virus initiates the coagulation pathway, which is also thought to be adding to the hypercoagulability [5]. As mentioned in the literature, the incidence of CVST in the pediatric age group is around 67%, with a preference for the male gender [6]. The common presenting complaints vary from signs of raised intracranial pressure like headache and vomiting to seizures and focal neurological deficits [6]. Clinically suspicious CVST can be further diagnosed with imaging techniques such as CT and MRI or MRV. The magnetic resonance studies are generally more sensitive as compared to the other studies [7]. Our patient presented with AMS associated with headaches and vomiting leading to CVST being one of the differentials, which the MRI eventually confirmed with MRV. Early and prompt thrombolysis with anticoagulation is the most commonly incorporated treatment. Low molecular weight heparin (LMWH) is the preferred treatment in the case of acute CVST. Endovascular thrombolysis is suggested in patients who are unresponsive to oral anticoagulants. Symptomatic therapy with anti-epileptic drugs is preferred for presenting complaints like seizures [8]. As per the latest guidelines, anticoagulation therapy was recommended for three to six months for a known cause and six to twelve months for an unknown cause of stroke [9]. Our patient responded very well to the anticoagulation therapy and was followed up further for any recurrence of his symptoms.

## Conclusions

In a nutshell, CVST is a rare extrapulmonary manifestation of COVID-19 infection and is extremely rare in the pediatric age group. It presents with different neurologic manifestations, and diagnosis is confirmed by imaging such as MRV and treated with anticoagulants.
